# The Interaction between Mitochondrial Oxidative Stress and Gut Microbiota in the Cardiometabolic Consequences in Diet-Induced Obese Rats

**DOI:** 10.3390/antiox9070640

**Published:** 2020-07-21

**Authors:** Adriana Ortega-Hernández, Ernesto Martínez-Martínez, Ruben Gómez-Gordo, Natalia López-Andrés, Amaya Fernández-Celis, Beatriz Gutiérrrez-Miranda, María Luisa Nieto, Teresa Alarcón, Claudio Alba, Dulcenombre Gómez-Garre, Victoria Cachofeiro

**Affiliations:** 1 Vascular Biology and Microbiota Laboratory, Hospital Clínico San Carlos-Instituto de Investigación Sanitaria San Carlos (IdISSC), 28040-Madrid, Spain; a.ortega.hernandez@hotmail.com (A.O.-H.); ruben.gomezgordo@gmail.com (R.G.-G.); 2 Ciber de Enfermedades Cardiovasculares (CIBERCV), Instituto de Salud Carlos III, 28029-Madrid, Spain; ernmarti@ucm.es (E.M.-M.); ml.nieto@csic.es (M.L.N.); 3 Departamento de Fisiología, Facultad de Medicina, Universidad Complutense de Madrid and Instituto de Investigación Sanitaria Gregorio Marañón (IiSGM), 28007 Madrid, Spain; 4 Cardiovascular Translational Research, Navarrabiomed, Complejo Hospitalario de Navarra (CHN), Universidad Pública de Navarra (UPNA), IdiSNA, 31008 Pamplona, Spain; natalia.lopez.andres@navarra.es (N.L.-A.); amaya.fernandez.decelis@navarra.es (A.F.-C.); 5 Instituto de Biología y Genética Molecular, CSIC-Universidad de Valladolid, 47003 Valladolid, Spain; bgutimiranda@gmail.com; 6 Servicio de Microbiología, Hospital Universitario de La Princesa, Instituto de Investigación Sanitaria La Princesa, Departamento de Medicina Preventiva, Salud Pública y Microbiología, Facultad de Medicina, Universidad Autónoma de Madrid, 28006 Madrid, Spain; talarcon@helicobacterspain.com; 7 Sección Departamental de Farmacia Galénica y Tecnología Alimentaria, Facultad de Veterinaria, Universidad Complutense de Madrid, 28040-Madrid, Spain; claudioalbarubio@gmail.com

**Keywords:** cardiac fibrosis, insulin resistance, microbiota, mucins, obesity

## Abstract

Background: The objective of this study is to determine the role of mitochondrial oxidative stress in the dysbiosis associated with a high fat diet in rats. In addition, the impact of gut microbiota (GM) in the cardiometabolic consequences of diet-induced obesity in rats has been evaluated. Methods: Male Wistar rats were fed either a high fat diet (HFD) or a control (CT) one for 6 weeks. At the third week, one-half of the animals of each group were treated with the mitochondrial antioxidant MitoTempo (MT; 0.7 mgKg^−1^day^−1^ i.p). Results: Animals fed an HFD showed a lower microbiota evenness and diversity in comparison to CT rats. This dysbiosis is characterized by a decrease in *Firmicutes/Bacteroidetes* ratio and relevant changes at family and genera compared with the CT group. This was accompanied by a reduction in colonic mucin-secreting goblet cells. These changes were reversed by MT treatment. The abundance of certain genera could also be relevant in the metabolic consequences of obesity, as well as in the occurrence of cardiac fibrosis associated with obesity. Conclusions: These results support an interaction between GM and mitochondrial oxidative stress and its relation with development of cardiac fibrosis, suggesting new approaches in the management of obesity-related cardiometabolic consequences.

## 1. Introduction

Nutrition is a key modulator of oxidative stress in the human body. Even under physiological conditions, nutrient intake is accompanied by a postprandial oxidative stress, with mitochondria being the major source of reactive oxygen species (ROS) [1]. Mitochondrial ROS production plays an important role in cell signaling and homeostasis. However, an excessive oxidative stress production could cause mitochondrial dysfunction and trigger cell damage and death by affecting its structures [2,3,4]. Clinical and experimental studies have demonstrated that mitochondrial dysfunction can participate in the deleterious consequences of several pathological conditions, including obesity [5,6,7]. In this sense, we have previously reported that mitochondrial ROS participate in the development of myocardial fibrosis associated with obesity, which can lead to diastolic dysfunction and could consequently promote heart failure [6,8]. Obesity is also associated with alterations in glucose homeostasis, in which mitochondrial ROS can also participate and that can further facilitate the cardiovascular complications in the context of obesity [9,10]. 

The gastrointestinal (GI) system contains a complex and active population of microorganisms known as the gut microbiota (GM), which can contribute to a variety of host processes, from energetic metabolism to modulation of the immune system [11,12]. The mechanisms regulating microbiota composition and diversity are multifactorial, with diet being a major driver [12,13]. Numerous studies have demonstrated a link between microbiota and obesity, as well as between insulin resistance and diabetes [14,15]. Obese subjects show reduced richness and diversity of GM, with an alteration in the specific pattern of the main phyla found in humans and rodents, *Firmicutes* and *Bacteroidetes* [16,17]. 

Different data have shown that GM can promote storage of calories as fat, thereby influencing the development and maintenance of obesity through different mechanisms, which includes the production of bacterial metabolites [11,16]. In addition, alterations in intestinal barrier increase the permeability to bacterial metabolites that can reach the circulation and induce the synthesis of proinflammatory cytokines that influence the function of distal organs [18,19]. It is well-known that obesity is associated with a low-grade inflammatory stage. 

A crosstalk between mitochondria and GM has been suggested. Mitochondria and microbiota not only share many structural and functional activities, but mitochondria can also affect the microbiome diversity and microbiota can affect mitochondrial function. This interaction can occur at different levels and mechanisms, including oxidative stress, and may be crucial for human health [20,21]. Several studies have reported that mitochondria ROS modulate the gut epithelial barrier, thereby influencing microbiot ROS [20]. By contrast, metabolites produced by GM modulate mitochondrial energy metabolism and activities [20,22,23]. Interestingly, diet could be an important modulator of mitochondria function, since the production of metabolites by microbiota is dependent on dietary compounds [12,13]. Therefore, the main purpose of this study was to evaluate whether mitochondrial oxidative stress can affect cardiometabolic consequences of diet-induced obesity through the modulation of the GM composition. To address this aim, we evaluated the impact of a high fat diet (HFD) on fecal microbiota composition and whether this effect could be modified by the administration of a mitochondrial targeted antioxidant. In addition, the interactions between GM and cardiac fibrosis and insulin resistance observed in obese rats were evaluated. 

## 2. Methods

This study was performed following the Animal Care and Use Committee of Universidad Complutense of Madrid and Dirección General de Medio Ambiente, Comunidad de Madrid, which approved all experimental procedures according to the Spanish Policy for Animal Protection RD53/2013, which meets the European Union Directive 2010/63/UE (PROEX 242/15).

### 2.1. MitoTempo Administration

The mitochondrial targeted antioxidant MitoTempo (MT) was obtained from Merck Sigma Aldrich (St. Louis, MO, USA). MT treatment was administered i.p once a day at the dose of 0.7 mg/Kg from the third week on. The MT dose was chosen based on a previous publication [8]. 

### 2.2. Animals and Experimental Groups

Male Wistar rats of 150 g, purchased from Envigo (Barcelona, Spain), were fed either a HFD (HFD, 35% fat; Envigo Teklad #TD.03307, Haslett, MI, USA; *n* = 16) or a standard diet (CT, 3.5% fat; Envigo Teklad #TD.2014; *n* = 16) for 6 weeks. Half of the animals of each group received the mitochondrial antioxidant MT. Therefore, 4 experimental groups were included in the study: CT (*n* = 8 animals), MT (*n* = 8 animals), HFD (*n* = 8 animals) and HFD + MT (*n* = 8 animals). All animals were held in a light- and temperature-controlled room with free access to diet and tap water. Food, water intake and weight were periodically controlled throughout the experimental period. At the end of the study, fasted animals were euthanized, and blood, white adipose tissue pads, heart, colon and fecal content were collected. For each animal, adiposity index was calculated as the sum of white fat pads/[(body weight-fat pad weight) × 100] [24].

### 2.3. Blood Biochemistry

Plasma glucose concentration was determined using an automatic analyzer (Vitros 5600, OrthoClinical). Plasma insulin levels were measured using a specific quantitative sandwich enzyme immunoassay (Mercodia, Uppsala, Sweden) according to the manufacturer’s instructions, and peripheral insulin sensitivity was calculated based on the Homeostasis Model Assessment (HOMA) [25]. 

### 2.4. Western Blotting

Total proteins were prepared as previously described [6] from cardiac homogenates from obese and control rats. Proteins were separated by SDS-PAGE on 12.5% polyacrylamide gels and transferred to polyvinylidene difluoride membranes (Hybond-P; Amersham Biosciences, Piscataway, NJ, USA). Membranes were probed with primary antibodies for α-smooth muscle actin (α-SMA; dilution: 1:500; Merck Sigma Aldrich), fibronectin (dilution: 1:500; Merck Millipore, Darmstad, Germany), periostin (dilution: 1:1000; Santa Cruz Biotechnology, Dallas, TX, USA) and vimentin (dilution 1:1000, Santa Cruz Biotechnology). Stain free detection was used for loading control (Bio-Rad Laboratories, Hercules, CA, USA). Results are expressed as an n-fold increase over the values of the control group in densitometric arbitrary units. 

### 2.5. Mucin Level Analysis in Goblet Cells

Colon tissue samples were dehydrated, embedded in paraffin and cut into 5 μm-thick sections. Mucin levels in goblet cells were quantified in Alcian Blue (AB)/periodic acid-Schiff (PAS)-stained sections using ImageJ image analysis program. Acidic mucins stain blue with AB and neutral mucins stain pink with PAS, while mixtures of neutral and acidic mucins appear purple. For each sample, 5 to 7 fields with at least ten complete contiguous colon crypts were analyzed with a 20X objective under transmitted light microscope. The area occupied by mucins was identified as the ratio of percentage of mucin content to the total tissue area. A single researcher unaware of the experimental groups performed the analysis.

### 2.6. Isolation of Microbial DNA

Microbial DNA was extracted from 150 mg of the fecal content from each animal using the QIAamp DNA Stool kit (Qiagen, Hilden, Germany) according to the manufacturer’s instructions. DNA integrity was evaluated with an Agilent 2100 Bioanalyzer System and DNA concentration was determined with a Qubit 3.0 Fluorometer using the dsDNA HS (High Sensitivity) Assay (Thermo Fisher Scientific, Madrid, Spain).

### 2.7. Amplification and Sequencing of the 16S rDNA Gene

For each sample, bacterial 16S rDNA was amplified by polymerase chain reaction (PCR) using the Ion 16S™ Metagenomics Kit (Thermo Fisher Scientific) that uses two primer pools to amplify seven hypervariable regions (V2, V4, V8 and V3, V6-7, V9, respectively). The amplification protocol was as follows: 95 °C for 10 min followed by 25 cycles of 95 °C for 30 s, 58 °C for 30 s and 72 °C for 20 s, and a final step of 7 min at 72 °C. Equal volumes of the two primer reactions were pooled and PCR amplicons purified with paramagnetic beads technology (CleanPCR, Labclinics, Barcelona, Spain). Barcoded libraries were prepared from 5 ng of DNA per sample using the Ion Plus Fragment Library Kit (Thermo Fisher Scientific) to end-repair amplicons and the Ion Xpress™ Barcode Adapters Kit (Thermo Fisher Scientific) to ligate the barcode adapters, according to the manufacturer’s instructions. Libraries were diluted to 22 pM prior to clonal amplification by emulsion PCR with the Ion OneTouch™ 2 System using the Ion 520™ and Ion 530™ Kit-OT2 (Thermo Fisher Scientific), and then sequenced on an Ion S5 System using a Ion 520™ Chip (Thermo Fisher Scientific).

### 2.8. Bioinformatic Analysis

Base calling, low quality filtering, removal of polyclonal reads and demultiplexing were automated by Torrent Suite™ software (v5.10.0; Thermo Fisher Scientific). Primer regions were removed and sequences were trimmed to 150 bp using self-developed Python scripts. FASTQ files were analyzed using QIIME 2 software (v2020.2) to dereplicate reads and remove singletons. SILVA 16S rRNA gene database (v132) was used to perform a reference-based clustering of operational taxonomic units (OTUs) at 99% similarity. OTUs with fewer than 10 observations across all samples were discarded, and relative OTU abundances were computed as percent proportions based on the total number of reads per sample.

Alpha and beta-diversity analyses were performed from the filtered OTU table to avoid any biases caused by the presence of rare OTUs using self-developed Python and R scripts. To assess alpha diversity, four metrics were calculated: observed OTUs, Chao1 richness estimate, Shannon diversity index and Pielou’s evenness index. 

To perform beta diversity analysis, Bray–Curtis dissimilarity and Jensen–Shannon divergence (JSD) distance matrices were computed. Principal coordinate analysis (PCoA) was applied to these metrics and the compositional dissimilarity between groups and samples was visualized in two-dimensional PCoA plots. 

Taxonomic assignment of OTUs was carried out using QIIME 2 and SILVA 16S taxonomy. Picrust2 software (v2.3.0-b) was used to predict the functional profiles of microbial communities in terms of Kyoto Encyclopedia of Genes and Genomes (KEGG) functional orthologs and metabolic pathways. Linear discriminant analysis (LDA) effect size (LEfSe) analysis was performed to differentially identify abundant taxa and functional features among groups. Taxa with a relative abundance less than 0.01% were filtered for this analysis. 

### 2.9. Statistical Analysis

Continuous variables are expressed as mean ± standard error of the mean (SEM). Normality of distributions was verified by means of the Kolmogorov–Smirnov test. Data normally distributed were analyzed using a one-way analysis of variance, followed by a Newman–Keuls to assess specific differences among groups or conditions. The differences in alpha and beta diversity metrics among groups were assessed using the non-parametric Kruskal–Wallis test, and the permutation-based multivariate analysis of variance (PERMANOVA) with 999 permutations, respectively. LEfSe analysis consists of the application of a Kruskal–Wallis test, followed by an LDA step, which assigns to each feature an LDA score that assesses its association with the categorical variables of interest. A threshold of *α* = 0.05 was considered statistically significant, establishing an LDA score cut-off of 3.0 for taxa and 2.0 for functional features. Association between genera relative abundances and cardiac collagen and HOMA index was studied using Pearson’s correlations. Genera showing |r| < 0.6 in their correlation with all the clinical parameters were filtered out. Multivariate analysis, considering either cardiac fibrosis or HOMA as the dependent variable, was performed with a linear regression model by means of a backward stepwise method. In consecutive steps, variables that were statistically significant in the univariable analysis were included in the linear regression model. Data analysis was performed using Python scripts and the statistical program SPSS v25.0 (SPSS Inc, Chicago, IL, USA). 

## 3. Results

### 3.1. Effects of MitoTempo in Metabolic and Cardiac Parameters of Diet-Induced Obese Rats

**Figure 1:**
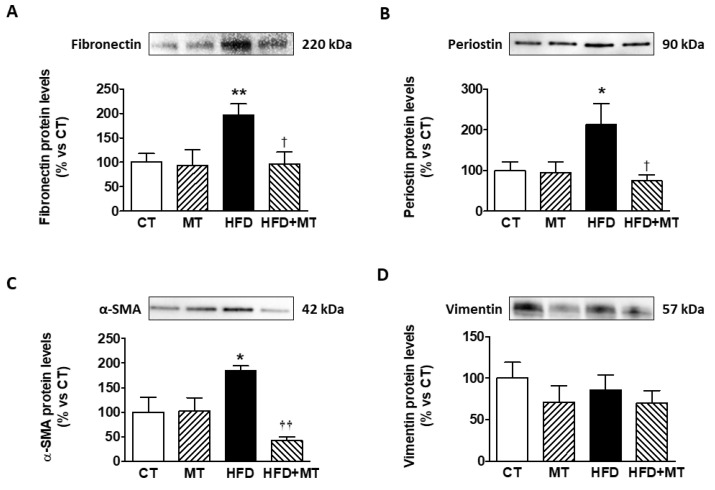
Protein levels of (**A**) fibronectin, (**B**) periostin, (**C**) α-smooth muscle actin (SMA) and (**D**) vimentin in heart from control rats fed a normal chow (CT) and rats fed a high fat diet (HFD) treated with vehicle or with the mitochondrial antioxidant MitoTempo (MT; 0.7 mg/Kg/day i.p). Bars graphs represent the mean ± SEM of 6–8 animals. Protein densitometry was expressed in arbitrary units (AU) once normalized to stain-free gel for protein.* *p* < 0.05; ** *p* < 0.01 vs. CT group. ^†^ *p* < 0.05, ^††^ *p* < 0.01 vs. HFD group.

The administration of the mitochondrial antioxidant MT to HFD rats reduced the increase in adiposity, and in the following metabolic parameters: fasting glucose, insulin levels and, in consequence, HOMA index showed by HFD-induced obese rats (Table 1). In addition, HFD animals receiving MT showed a significant decrease in relative heart weight and cardiac interstitial fibrosis [8], but also α-SMA, fibronectin and periostin protein expression (Figure 1) as compared with HFD rats treated with vehicle. MT did not affect any of these parameters in CT animals (Table 1 and Figure 1A–D).

### 3.2. Effects of MitoTempo in the Mucin Levels in the Colon of Diet-Induced Obese Rats 

**Figure 2:**
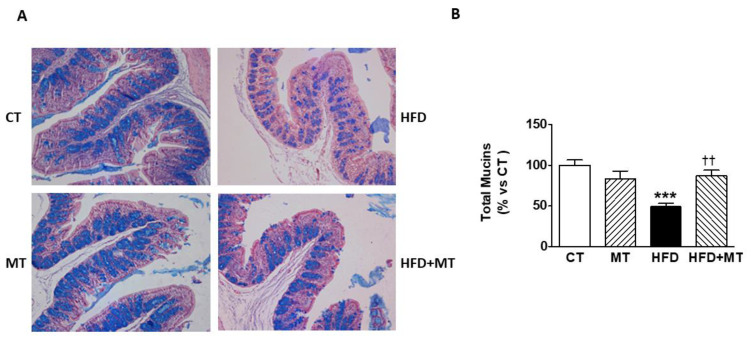
(**A**) Representative microphotographs and (**B**) quantification of total mucin levels in colon from control animals fed a normal chow (CT) and animals fed a high fat diet (HFD) treated with vehicle or with the mitochondrial antioxidant MitoTempo (MT; 0.7 mg/Kg/day i.p) stained with Alcian Blue (AB)/periodic acid-Schiff (PAS) examined by light microscopy (magnification 20×). Bar graphs represent the mean ± SEM of 5–6 animals normalized to for CT group. *** *p* < 0.001 vs. CT group; ^††^ *p* < 0.01 vs. HFD group.

We used the combined analysis of AB/PAS staining to evaluate both neutral and acidic mucin secreting cells in colon simultaneously. As shown in Figure 2, there is a predominance of goblet cells containing acidic mucins over neutral mucins in the rat colon, in a proportion ~3:1, in CT rats fed a standard diet. HFD rats showed a significant drop-off in the overall AB/PAS staining when compared with CT animals that was reversed by MT (Figure 2A,B). Since MT neither affected the levels of colonic goblet cells containing neutral and acidic granules in CT animals (Figure 2) nor metabolic parameters (Table 1) or cardiac proteins (Figure 1), to simplify, only data from CT, HFD or HFD+MT groups will be presented from now on.

### 3.3. Effects of MitoTempo in Gut Microbiota Diversity of Diet-Induced Obese Rats

Fecal DNA was isolated from 8 CT rats, 8 HFD rats and 7 HFD rats treated with MT. After 16S rDNA sequencing, 6,332,097 reads were successfully classified into 6119 OTUs that were used for downstream analysis.

First, we studied the alpha diversity of GM, which refers to the intrinsic biodiversity of the samples, in CT, HFD and HFD+MT rats. No differences were found in richness among any group, as assessed by observed OTUs and Chao1 indexes (Table 2). By contrast, we observed that Shannon and Pielou’s indexes were significantly reduced in HFD rats, thus revealing lower microbiota evenness and overall diversity in comparison to control rats. The administration of MT was able to reverse these changes (Table 2). 

Next, we estimated beta diversity of GM in CT, HFD and HDF+MT rats. Bray–Curtis dissimilarity and JSD indexes revealed significant differences between HFD group as compared with CT group. MT partially, but significantly, restored GM composition, although it was not normalized since significant differences were still observed between MT-treated animals and CT ones (Table 2). 

### 3.4. Effects of MitoTempo in Gut Microbiota Composition of HFD-Induced Obese Rats

**Figure 3:**
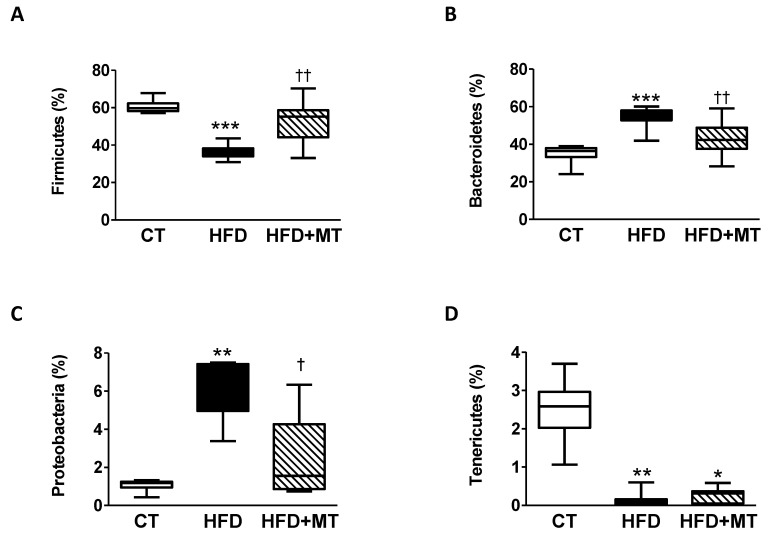
Boxplot showing total relative abundance of reads of the four most abundant taxa at Phylum level in the gut microbiota (**A**) *Firmicutes*, (**B**) *Bacteroidetes*, (**C**) *Protobacteria* and (**D**) *Tenericutes* in feces from control animals fed a normal chow (CT) and animals fed a high fat diet (HFD) treated with vehicle or with the mitochondrial antioxidant MitoTempo (MT; 0.7 mg/Kg/day i.p). Upper, middle and lower lines represent first quartiles, medians and third quartiles. The whiskers represent a 1.5 * inter-quartile range. Data are expressed as percentage of total reads. * *p* < 0.05; ** *p* < 0.01; *** *p* < 0.001 vs. CT group. ^†^ *p* < 0.05, ^††^ *p* < 0.01 vs. HFD group.

Taxonomic assignment of OTUs gave rise to the identification of 15 phyla, 25 classes, 42 orders, 79 families and 275 genera in the GM. In all groups, the most abundant phyla were *Firmicutes*, *Bacteroidetes*, *Proteobacteria* and *Tenericutes*, although with significant differences in their abundances among groups (Figure 3A–D). GM composition in HFD rats was more abundant in *Bacteroidetes* and *Proteobacteria*, and less in *Firmicutes* and *Tenericutes* when compared to CT animals. The administration of MT to HFD rats normalized the abundance of bacteria belonging to phyla *Firmicutes, Bacteroidetes* and *Proteobacteria.* However, it did not affect the reduction in *Tenericutes* observed in HFD rats (Figure 3A–D).

**Figure 4:**
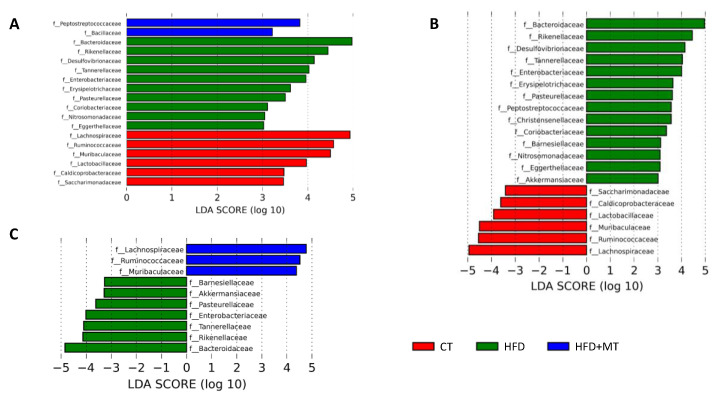
LEfSe analysis showing taxonomic differential abundance at family level in feces from control animals fed a normal chow (CT) and animals fed a high fat diet (HFD) treated with vehicle or with the mitochondrial antioxidant MitoTempo (MT; 0.7 mg/Kg/day i.p). (**A**) Significantly different families among CT, HFD and HFD+MT groups. (**B**) Significantly enriched and depleted families between CT and HFD groups. (**C**) Significantly enriched and depleted families between HFD and HFD + MT groups. The length of the horizontal bars represents the LDA score (effect size). *p* < 0.05; LDA score > 3.0.

LEfSe analysis of bacterial taxa constituting GM, at the family level, is shown in Figure 4. We found that 6 families characterized the CT group: 3 belonged to the *Firmicutes* phylum (*Lachnospiraceae, Ruminococcaceae* and *Lactobacillaceae*); 1 to the *Bacteroidetes* phylum (*Muribaculaceae*), 1 to the *Proteobacteria* phylum (*Caldicoprobacteraceae*) and 1 to the *Patescibacteria* phylum (*Saccharimonadaceae*) (Figure 4A). Obese animals showed a decrease in all these families with respect to CT (Figure 4B). By contrast, obesity was accompanied by an enrichment of 14 families with respect to CT group (Figure 4B). Consistently with phylum composition: 4 families belonged to the *Bacteroidetes* phylum (*Bacteroidaceae, Rikenellaceae*, *Barnesiellaceae* and *Tannerellaceae*); 4 to the *Proteobacteria* phylum (*Desulfovibrionaceae, Enterobacteriaceae, Pasteurellaceae* and *Nitrosomonadaceae*), 3 to the *Firmicutes* phylum (*Peptostreptococcaceae*, *Christensenellaceae* and *Erysipelotrichaceae*), 2 to the *Actinobacteria* phylum (*Coriobacteriaceae, Eggerthellaceae*) and 1 to the *Verrucomicrobia* phylum (*Akkermansiaceae*). Ten of these families characterized the HFD group: *Bacteroidaceae* and *Rikenellaceae*, and *Desulfovibrionaceae, Enterobacteriaceae, Pasteurellaceae and Nitrosomonadaceae* from *Bacteroidetes* and *Proteobacteria* phyla, respectively, and *Erysipelotrichaceae* and *Coriobacteriaceae,* and *Eggerthellaceae* from *Firmicutes* and *Actinobacteria* phyla, respectively (Figure 4A). MT treatment was able to reverse the abundance of some families: it increased the diminution of *Lachnospiraceae, Ruminococcaceae* and *Muribaculaceae* observed in HFD rats, and decreased the rise in the number of families of *Bacteroidetes* (*Bacteroidaceae, Rikenellaceae, Barnesiellaceae* and *Tannerellaceae*), and *Verrucomicrobia* phylum (*Akkermansiaceae*), and in some families of *Proteobacteria* (*Enterobacteriaceae* and *Pasteurellaceae*) (Figure 4C). Two families belonging to *Firmicutes* phylum characterized the group of HFD animals treated with MT: *Peptostreptococcaceae* and *Bacillaceae*.

**Figure 5:**
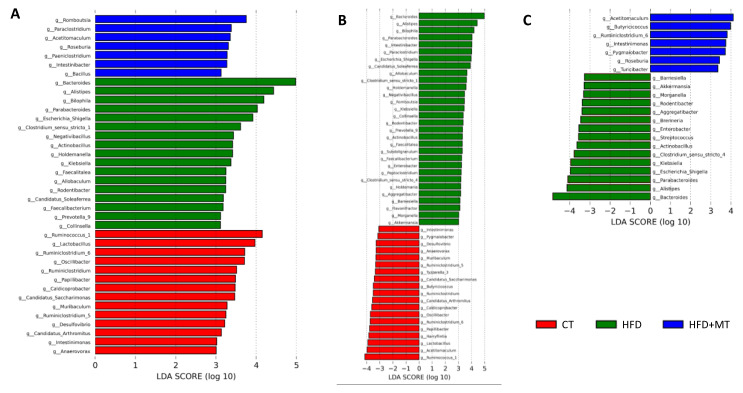
LEfSe analysis showing taxonomic differential abundance at genus level in feces from control animals fed a normal chow (CT) and animals fed a high fat diet (HFD) treated with vehicle or with the mitochondrial antioxidant MitoTempo (MT; 0.7 mg/Kg/day i.p). (**A**) Significantly different genera among CT, HFD and HFD+MT groups. (**B**) Significantly enriched and depleted genera between CT and HFD groups. (**C**) Significantly enriched and depleted genera between HFD and HFD + MT groups. The length of the horizontal bars represents the LDA score (effect size). *p* < 0.05; LDA score > 3.0.

At the genus level, GM of CT and HFD rats showed 14 and 17 genera differentially found, respectively, while obese animals treated with MT were characterized by 7 genera (Figure 5A). Regarding genera driving divergences between groups, 30 genera were enriched in HFD animals with respect to CT animals: 16 belonged to the *Firmicutes* phylum, 4 to the *Bacteroidetes* phylum (*Bacteroides* presented the highest difference), 8 to the *Proteobacteria* phylum and 1 to the *Verrucomicrobia and Actinobacteria* phyla (Figure 5B). By contrast, 19 genera were reduced in HFD animals as compared with controls, 14 belonging to *Firmicutes* phylum (*Ruminococcus 1* presented the highest difference), 2 to *Proteobacteria* and 1 to *Bacteroidetes* and *Saccharibacteria* (Figure 5B). MT treatment was able to reverse only some changes of the GM composition of obese animals. It increased the abundance of 7 genera out of 19 (6 genera belonging to *Firmicutes* phylum, with *Acetitomaculum* presenting the highest difference) and a single genus belonging to *Actinobacteria* (*Pygmaiobacter*). It reduced the content of 15 genera out of 30 (8 belonging to *Proteobacteria* phylum, 3 belonging to *Bacteroidetes,* with *Bacteroides* presenting the highest difference, 3 to *Firmicutes* and a single genus to *Verrucomicrobia* phylum (Figure 5C)).

### 3.5. Relationship between Genera Abundance and Metabolic and Cardiac Parameters

Relative abundances of all identified genera were correlated with cardiac collagen levels, HOMA and adiposity indexes. Among genera displaying a significant correlation with these parameters, *Morganella* showed the highest correlation with cardiac collagen (Pearson’s r = 0.831); *Candidatus soleaferrea* with HOMA (Pearson’s r = 0.791) and *Muribaculum* with adiposity (Pearson’s r = −0.761). Multivariate analysis showed that 1 genus from *Proteobacteria* phylum (*Morganella*) and 4 from *Firmicutes* phylum (*Eubacterium oxidoreducens group*, *Lachnospiraceae ND3007 group, Erysipelotrichaceae UCG-003, Ruminococcus 1* and *Streptococcus*) were independent predictors of collagen content (Table 3).

One genus from Bacteroidetes (uncultured Muribaculaceae) and 4 from Firmicutes (Candidatus soleaferrea, Peptoclostridium, Eubacterium oxidoreducens group and Holdemania were independent predictors of HOMA index (Table 3).

### 3.6. Effects of MitoTempo in Gut microbiota Metabolism of Diet-Induced Obese Rats

**Figure 6:**
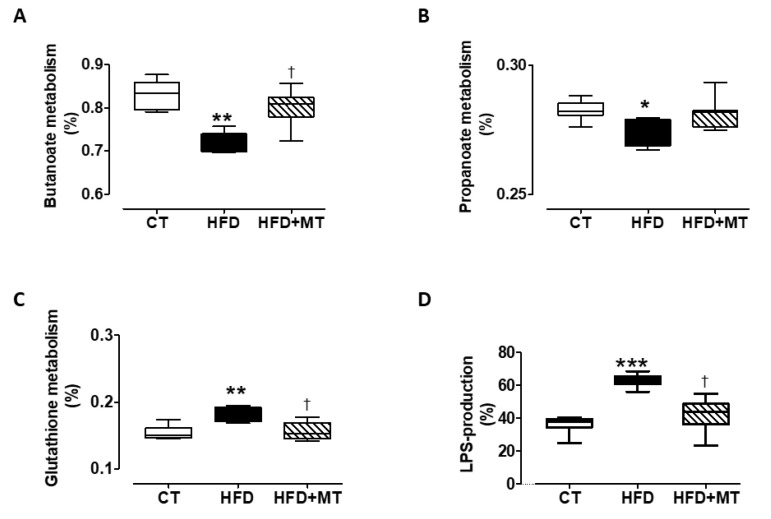
Boxplot showing total relative abundance of metabolic pathways related to gut microbiota in (**A**) butanoate metabolism, (**B**) propanoate metabolism and (**C**) glutathione metabolism and (**D**) percentage of bacteria involved in lipopolysaccharyde (LPS) production in feces from control animals fed a normal chow (CT) and animals fed a high fat diet (HFD) treated with vehicle or with the mitochondrial antioxidant MitoTempo (MT; 0.7 mg/Kg/day i.p). Upper, middle and lower lines represent first quartiles, medians and third quartiles. The whiskers represent a 1.5 * inter-quartile range. Data are expressed as percentage of total reads. * *p* < 0.05; ** *p* < 0.01; *** *p* < 0.001 vs. CT group. ^†^ *p* < 0.05 vs. HFD group.

To identify metabolic pathways significantly different among rat groups, a LEfSe analysis was performed. Obesity was accompanied by a reduction in the pathways related to butanoate (ko00650) and propanoate (ko00640) metabolism, which were improved in those animals treated with MT (Figure 6A,B). By contrast, an increase in the abundance of genera involved in glutathione metabolism (ko00480) was observed in HFD-induced obese animals as compared with control ones, which was normalized by treatment with the mitochondrial antioxidant (Figure 6C). An increase in abundant taxa of genera involved in lipopolysaccharide (LPS) production (ko00540) was found in HFD as compared with CT, and MT was able to normalize it (Figure 6D).

## 4. Discussion

Obesity is closely associated with GM dysbiosis. We have previously shown the relevance of mitochondrial oxidative stress in cardiac alterations associated with obesity [8]. In this study, we have now demonstrated that the administration of the mitochondrial antioxidant MT prevents HFD diet-induced obesity, insulin resistance and cardiac fibrosis in association with a preservation of mucin- goblet cells in colon and a restoration of GM composition. Interestingly, we observed that changes in the abundance of certain genera could be relevant not only in the development of obesity and its metabolic consequences but also in the occurrence of cardiac fibrosis associated with obesity. 

As already reported, HFD rats show a different gut environment with respect to lean animals characterized by low diversity and richness of gut microbiome, a feature associated with obesity and its metabolic alterations [26,27]. As in humans, the two dominant phyla observed in both lean and obese rats are *Firmicutes* and *Bacteroidetes,* although with significant differences about their relative distributions among the studies [28]. This variety in the results may rely, at least in part, on different methodological approaches (metagenomics sequencing, sample microbial load) but also in the area of the gut from which the samples were taken [16,29]. In this study, HFD rats showed a low *Firmicutes/Bacteroidetes* ratio with elevation of the *Bacteroidetes* phylum and decreasing levels of the *Firmicutes* phylum. Bacteria belonging to *Firmicutes* are important producers of short-chain fatty acids (SCFAs) and have been associated with healthy intestinal barrier and a reduction in endotoxin leakage [30,31,32]. *Bacteroidetes*, the largest phylum of Gram-negative, can release LPS involved in inflammatory activation, mainly when the intestinal mucosal barrier is disrupted. In this sense, obese animals showed a reduction in mucin-secreting goblet cells that may result in a defective mucin production, pointing to a damaged intestinal barrier.

GM dysbiosis has been associated with insulin resistance, a common alteration observed in the context of obesity [14,33]. Our data show that 5 genera were independent predictors of HOMA (an index of insulin resistance), 2 of them in a positive manner (*Candidatus soleaferrea* and *Peptoclostridium*) and 3 in a negative one (*uncultured Muribaculaceae, Eubacterium oxidoreducens group* and *Holdemania*). Different data have also reported the association between insulin resistance and the levels of these genera [14,34,35,36]. 

Fibrosis is a well-known feature of the cardiac remodeling associated with obesity that can favor functional alterations by reducing cardiac relaxing capability and therefore increase its filling pressure. Our data show that the fibrosis observed in obese animals not only involved an increase in collagen content [8], the main component of extracellular matrix (ECM), but also of other components of ECM such as periostin and fibronectin. Of special interest, periostin is a potent modulator of cell–matrix interaction that has been implicated in the crosstalk between multiple signaling pathways including TGF-β [37]. In line with our results, treatment of diabetic animals with the antioxidant resveratrol resulted in a significant reduction in myofibroblast activation, and inhibited the expression of periostin via TGF-β signaling [38]. Concerning fibronectin, its blockade has been described as attenuating cardiac fibrosis in an experimental model of heart failure [39]. This excessive ECM deposit was associated with an increase in myofibroblasts as indicated by the increased α-SMA levels, the cell mainly responsible for fibrosis. The analysis show that 6 genera were independent predictors of fibrosis content, both in positive (*Morganella* and *Streptococcus*) and in a negative manner (*Eubacterium oxidoreducens group, Lachnospiraceae ND3007 group, Erysipelotrichaceae UCG-003* and *Ruminococcus 1*). In a model of cirrhosis in rats, the treatment with antibiotics induced an exacerbation of hepatic fibrosis that was associated with overgrowth by *Morganella morganii* [40]. Similarly, *Streptococcus* can trigger the progression of pulmonary fibrosis in two different mouse models of pulmonary fibrosis [41], supporting a profibrotic capacity of these genera. No information regarding the potential role in fibrosis has been reported about the genera negatively associated with cardiac fibrosis in our study. However, they are more abundant in healthy subjects than in subjects with different pathological conditions [42,43]. 

Several mechanisms could account for the beneficial effects induced by the mitochondrial anti-oxidant. The impact of this microbiota profile on the gut’s mucosal integrity and the translocation of microbial cell wall components, like LPS from Gram-negative bacteria and other toxic metabolites, could be the potential mechanisms involved in the impact of the gut dysbiosis in the cardiometabolic consequences in the context of obesity [19,40,42,43,44]. Dysbiosis can affect the integrity of the gut barrier, due to abnormal or reduced numbers of mucous cell production, resulting in diminished mucus production and both enhanced permeability and transit of antigens [45].

One of the hallmarks of obesity and obesity-related pathologies is the occurrence of chronic low-grade inflammation that may affect the fibrotic development [46]. This inflammation-related process can be initiated by LPS [47]. Our metabolic pathway analysis showed an increase in LPS-producing bacteria in the gut of obese rats that was inhibited by the anti-oxidant. Moreover, MT-treated obese rats also restored bacteria genera involved in the pathways of propanoate and butanoate metabolism, SCFA essential for the maintenance of intestinal epithelium physiology by regulating the cellular turnover and barrier functions. In addition, SCFA modulate the inflammatory response by suppressing the production of pro-inflammatory mediators induced by LPS or inducing differentiation of regulatory T cells [29,48,49].

Moreover, obese animals show an increase in bacteria genera involved in the pathways of glutathione metabolism that protects against oxidative stress, especially in the heart. Disturbances in cardiac glutathione homeostasis has been associated with cardiac lipotoxicity in diet-induced obesity in mice [50]. The fact that these alterations were reversed by MT further support a crosstalk between microbiota–mitochondria. 

In conclusion, our data demonstrated that the administration of a mitochondrial anti-oxidant to HFD obese rats modulates the GM composition, promoting a change in ratio *Firmicutes* to *Bacteroidetes*, thereby decreasing bacteria genera associated with insulin resistance, fibrosis and inflammation. These results further support an interaction between GM and mitochondrial oxidative stress, suggesting new approaches in the management of obesity-related cardiometabolic consequences. 

## Figures and Tables

**Table 1:** Effect of the mitochondrial antioxidant MitoTempo (MT; 0.7 mg/Kg/day i.p) on metabolic parameters in rats fed a normal chow (CT) and high fat diet (HFD) fed rats.

Variable	CT	MT	HFD	HFD + MT
Adiposity index (%)	3.49 ± 0.26	3.52 ± 0.22	8.39 ± 0.41 ***	6.9 ± 0.55 *^,^^†††^
Glucose (mg/dL)	98.8 ± 4.6	95.2 ± 2.8	114.6 ± 2.1 **	104.3 ± 2.5 ^†^
Insulin (pg/mL)	72.2 ± 7.7	60.9 ± 5.9	241.6 ± 30.8 ***	132.7 ± 16.5 *^,^^†††^
HOMA index	2.50 ± 0.33	1.98 ± 0.19	9.28 ± 1.1 ***	4.8 ± 0.7 *^,^^†††^

Data are mean ± SEM, *n* = 7–8 in each group. * *p* < 0.05; ** *p* < 0.01; *** *p* < 0.001 vs. CT group, ^†^ *p* < 0.05; ^†††^ *p* vs. HFD group.

**Table 2:** Alpha and beta diversity indexes in rats fed a normal chow (CT) and high fat diet (HFD) and treated with the antioxidant mitochondrial MitoTempo (MT; 0.7 mg/Kg/day i.p).

Variable	CT	HFD	HFD + MT
Observed OTUs	3267 ± 121	3495 ± 117	3458 ± 98
Chao1 richness index	3945 ± 100	4168 ± 1128	4095 ± 61
Shannon index	7.93 ± 0.14	7.51 ± 0.10 *	7.95 ± 0.14 ^†^
Pielou’s evenness index	0.68 ± 0.011	0.64 ± 0.01 *	0.68 ± 0.013 ^†^
Bray–Curtis dissimilarity	0.549 ± 0.017	0.423 ± 0.02 **	0.62 ± 0.041 *^,†^
JSD distance	0.483 ± 0.013	0.388 ± 0.015 **	0.521 ± 0.033 *^,†^

Jensen-Shannon divergence (JSD). Data are mean ± SEM, *n* = 7–8 in each group. * *p* < 0.05 vs. CT group; ** *p* < 0.01 vs. CT group; ^†^ *p* < 0.05 vs. HFD group.

**Table 3:** Multivariate models for collagen content and Homeostasis Model Assessment (HOMA) index.

Genus	Collagen Content		HOMA Index	
-	Β (95% CI)	*p*	Β (95% CI)	*p*
*Eubacterium oxidoreducens group*	−0.73 (−1.17 to −0.28)	0.003	−2.36 (−3.73 to −0.98)	0.002
*Streptococcus*	6.26 (0.30 to 12.22)	0.041		
*Lachnospiraceae ND3007 group*	−3.42 (−6.17 to −0.67)	0.018		
*Erysipelotrichaceae UCG-003*	−63.64 (−109.45 to −17.08)	0.01		
*Ruminococcus 1*	−0.13 (−0.25 to −0.11)	0.034		
*Morganella*	10.71 (6.91 to 14.51)	<0.0001		
*Muribaculaceae (uncultured bacterium)*			−0.457 (−0.667 to −0.246)	<0.0001
*Candidatus Soleaferrea*			253.77 (125.49 to 382.05)	0.001
*Holdmania*			−107.70 (−177.80 to −37.70)	0.005
*Peptoclostridium*			82.47 (5.56 to 159.38)	0.037
